# Moxibustion at ‘Danzhong’ (RN17) and ‘Guanyuan’ (RN4) for fatigue symptom in patients with depression: Study protocol clinical trial (SPIRIT Compliant)

**DOI:** 10.1097/MD.0000000000019197

**Published:** 2020-02-14

**Authors:** Somayeh Iravani, Liwei Cai, Lue Ha, Shuzhe Zhou, Chuan Shi, Yibin Ma, Qin Yao, Ke Xu, Baixiao Zhao

**Affiliations:** a School of Acupuncture-Moxibustion and Tuina, Beijing University of Chinese Medicine, Beijing 100029; b Peking University Sixth Hospital, Beijing 100191; c School of Traditional Chinese Medicine, Beijing University of Chinese Medicine, Beijing 100029, China.

**Keywords:** depression-related fatigue, moxibustion, randomized controlled trial, Traditional Chinese Medicine

## Abstract

**Background:**

Fatigue is one of the most prevalent and debilitating symptoms of major depressive disorder (MDD). The effective management of depression-related fatigue has an important impact on the patient's abilities, functioning, and quality of life (QOL). Moxibustion has been widely used in Traditional Chinese Medicine to manage fatigue. Recent studies have also demonstrated that moxibustion is effective for treating cancer-related fatigue and chronic fatigue syndrome. However, there is not sufficient data supporting the effect of moxibustion for depression-related fatigue. Therefore, this randomized, assessor-blinded, wait-list controlled trial is designed to evaluate the effectiveness, safety, and feasibility of moxibustion treatment for depression-related fatigue.

**Methods:**

One hundred and seventy-six participants who meet the diagnostic criteria for depression in the International Classification of Diseases, tenth revision (ICD-10), and who also have a score of ≥1 on the 13^th^ item of the Hamilton Depression Rating Scale-17 (HAMD-17), will be enrolled. At study entry, participants will undergo anti-depressant treatment for at least 1 month. Then those who still have a score of ≥1 on the 13^th^ item of the HAMD-17 will be randomly allocated to either a moxibustion group or wait-list control group in a ratio of 1:1. Anti-depressants will be provided for both groups during the whole process of the study period. Participants in the moxibustion group will undergo 14 sessions of moxibustion (over 2 weeks) with anti-depressant treatment, and participants in the wait-list control group will receive only anti-depressant treatment. Subsequently, participants in the moxibustion group will be followed-up for 4 weeks. The primary outcome measure will be the Fatigue Severity Scale (FSS). The secondary outcome measure will be the HAMD-17. Safety will be assessed by monitoring adverse events during the study. Trial feasibility will also be assessed in this study.

**Discussion:**

The results of this study may provide evidence for the efficacy of moxibustion as an adjunct to antidepressants for depression-related fatigue, and promote a more widespread foundation for the selection of moxibustion in the clinical setting as well as for future research in moxibustion therapy.

**Trial registration:**

This study protocol was registered at the Chinese Clinical Trial Registry (ChiCTR1800016905).

## 1 Introduction

Major depressive disorder (MDD) is recognized as one of the most disabling medical conditions, affecting approximately 322 million people worldwide.^[1]^ Moreover, it is estimated to become the second leading cause of global burden of disease by 2030.^[2]^ Among the many symptoms of MDD, fatigue is one of the most prevalent and debilitating symptoms,^[3–5]^ and occurs in more than 90% of patients.^[6]^ It is characterized by an overwhelming and persistent sense of tiredness, weakness or exhaustion and decreased capacity for mental and physical work.^[7]^ Fatigue has been shown to be a most prominent and bothersome residual symptom of depression and may persist in approximately 20–38% of patients who have achieved remission.^[8]^

Depression-related fatigue is observed in depression patients with various clinical statuses. It occurs prodromal to a depressive episode, within the course of a major depressive episode, as a residual symptom even when other depressive symptoms have remitted, or as a treatment-emergent side effect.^[6]^ Fatigue in patients with depression is most strongly associated with impaired work and social functioning,^[9,10]^ diminished health-related quality of life (QOL), and disability.^[4,6,11]^ Furthermore, residual fatigue is contributed to be a major risk factor for chronicity and relapse.^[12]^ In fact, physical impairment and disability from chronic fatigue and its negative economic consequences can cause direct and indirect costs of 17 and 24 billion dollars annually.^[13]^

The mechanism of depression-related fatigue has not yet been fully elucidated, but it has been reported to be associated with the dysregulation of neurotransmitters, such as norepinephrine, dopamine, acetylcholine, histamine, and serotonin in the cortex,^[14,15]^ a reduction of neuronal activity in prefrontal cortex,^[16]^ the dysregulation of the hypothalamic-pituitary-adrenal axis,^[17]^ and a dysfunction of the neuroimmune system including inflammatory cytokines.^[18]^

Several pharmacological agents, such as venlafaxine, bupropion, fluoxetine, and sertraline have been proposed to be the first-line treatment for depressed patients with prominent fatigue.^[14,19]^ In addition, combining stimulants or modafinil with these first-line treatments may provide a better effect in improving fatigue symptoms.^[20]^ However, at present no current Food and Drug Administration-approved treatments are available for fatigue.^[19]^ Therefore, more effective and safe alternative therapies for depression-related fatigue are needed by both patients and doctors.

Moxibustion, a modality of traditional acupuncture, is an external therapy that involves burning herbal preparations containing *Artemisia vulgaris* (mugwort) on or above the skin at acupoints, to warm them and alleviate symptoms.^[21–24]^ Moxibustion has been widely used for the prevention and treatment of various disorders in China for more than 2500 years,^[25,26]^ and has been especially applied to those who experience fatigue or similar conditions.^[26]^ Evidence from modern scientific studies also has demonstrated that moxibustion may be safe and effective for managing chronic fatigue syndrome,^[27,28]^ cancer-related fatigue,^[29,30]^ and exercise-induced fatigue.^[31]^ The exact mechanisms by which moxibustion appears to improve fatigue has not yet been fully understood. However, its mechanism has been reported to be related to changes of cytokine production,^[32]^ and improvement of inflammation,^[33]^ regulation of the hypothalamic-pituitary-adrenal axis, upregulating hippocampal progranulin expression,^[34]^ and reduction of oxidative damage.^[27,35]^

Although moxibustion has empirically been used for alleviating depression-related fatigue, to the best of our knowledge, there are no comprehensive clinical studies concerning the effects of moxibustion for the treatment of fatigue in patients with depression so far, underscoring the need for well-designed clinical trials to provide evidence for moxibustion in treatment of fatigue in depression patients, to improve function and QOL in the depression patients.

Since, previous research showed that moxibustion had good treatment effects on chronic fatigue syndrome, cancer related fatigue and exercise-induced fatigue, we hypothesized that moxibustion on depression-related fatigue will also have positive therapeutic effects. Therefore, this study is designed to investigate the effectiveness, safety, and feasibility of moxibustion treatment on depression-related fatigue.

## 2 Methods

### 2.1 Design and setting

**Figure 1:**
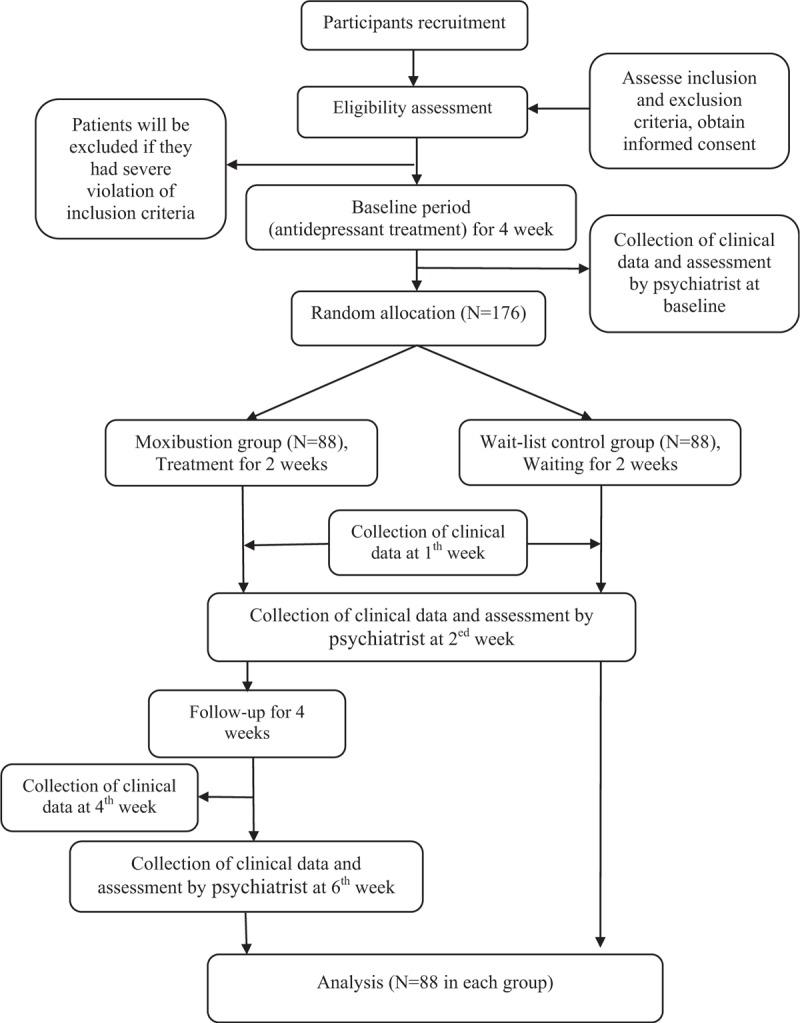
Flowchart of this study.

A prospective, randomized, wait-list controlled, trial was designed with cooperation between Beijing University of Chinese Medicine and Peking University Sixth Hospital and will be conducted in an outpatient department of Peking University Sixth Hospital, China, from June 2018 until December 2020. One hundred and seventy-six participants who meet the diagnostic criteria for depression in the International Classification of Diseases, tenth revision (ICD-10),^[36]^ and who also have the score of ≥1 on the 13^th^ item of the Hamilton Depression Rating Scale-17 (HAMD-17), will be enrolled. At study entry, participants will undergo antidepressant treatment for at least one month. Then, those who still have a score of ≥1 on the 13^th^ item of the HAMD-17 will be randomly allocated to either a moxibustion group or wait-list control group in a ratio of 1:1. Anti-depressants will be provided for both groups during the whole process of the study period. Participants in the moxibustion group will undergo 14 sessions of moxibustion (over 2 weeks) with anti-depressant treatment, while participants in the wait-list control group will receive only anti-depressant treatment. Subsequently, the moxibustion group will be followed up for 4 weeks. The flowchart of this trial is shown in Figure 1.

This study protocol has been approved by the Medical Ethics Committee Board of the Peking University Sixth Hospital (ethical approval number: 2018-13) and will be performed according to the guidance and principles of the Declaration of Helsinki. Informed written consent will be obtained from all of the participants before randomization.

### 2.2 Participants

Participants will be recruited from the Peking University Sixth Hospital through multimodal strategies, including outpatient clinics of the Peking University Sixth Hospital, posters on notice boards in this hospital with recruitment notices on the flyers, and social media (WeChat). Every potential participant with interest in the study will undergo a basic evaluation. Those who decide to participate, satisfy all the inclusion criteria, and provide written informed consent will be enrolled and will undergo further evaluation by psychiatrist and antidepressant treatment. Participants will return the next month for randomization if they still have a score of ≥1 on the 13^th^ item of the HAMD-17.

#### 2.2.1 Sample size

In this study, the sample size was calculated according to effective rate using PASS 11 software. It is generally considered that the difficulty in the treatment of fatigue symptoms is not lower than the average difficulty in treatment of all depression symptoms. Therefore, based on clinical experience and literature review,^[37,38]^ it is estimated that the success rates of anti-depressants and moxibustion on fatigue symptoms, are expected to be 50% and 80% respectively. With a 1% significance level (α = 0.01) and 90% power (1 − β = 0.9), and two group samples in a ratio of 1:1, the sample size was calculated to be 73. Considering a 20% drop-out rate, 88 participants are needed per group. In conclusion, a total of 176 participants are required in this trial.

#### 2.2.2 Inclusion criteria

Patients will be included if

(1) they are aged between 18 and 60 years,
(2) they fulfill the diagnostic criteria for depression in the ICD-10,^[36]^ including a single or recurrent episode,
(3) they have a score of ≥1 on the 13^th^ item of the Hamilton Depression Rating Scale (HAMD-17), and
(4) they also provide written informed consent.

#### 2.2.3 Exclusion criteria

Participants will be excluded if they

(1) have a diagnosis of any Axial 1 disorder in ICD-10 other than depression,
(2) have brain organic diseases, mental retardation, or a history of head trauma with a loss of consciousness for more than 5 minutes,
(3) have a current significant risk of suicide or serious agitation,
(4) have a history of receiving moxibustion treatment within the last 6 months, or
(5) women who are pregnant, lactating, or planning to become pregnant within the next 3 months.

#### 2.2.4 Withdraw criteria

Participants could withdraw from the study at any time due to any reason. This decision will not affect their treatment. Moreover, participants who have developed severe adverse events (AEs) will withdraw from the study and they will be monitored until its resolution.

### 2.3 Randomization

Eligible participants will be randomized in a 1:1 ratio to either a moxibustion group or wait-list control group by the Clinical Pharmacological Center of Peking University Sixth Hospital, using a computer-generated random allocation sequence in SPSS 20.0 software (IBM, Armonk, NY). The allocation sequence will be concealed from the researchers in sealed and sequentially numbered opaque envelopes and kept in a double-locked cabinet. The practitioners will open the envelopes and allocate the participants who meet the eligibility criteria after signing an informed consent and completing all baseline assessments. This procedure will ensure adequate randomization concealment.

### 2.4 Blinding

In this study, it is not feasible to blind the practitioner and participants due to the unique nature of moxibustion treatment. But treatment and evaluation will be performed independently. And outcome assessors, data collectors as well as the statistician will be blinded to group allocation throughout the study.

### 2.5 Interventions

During the treatment process, all participants regardless of their assignment will receive anti-depressant treatment. Participants who start a new regimen during the study period that might affect their fatigue severity, will be excluded from the study.

#### 2.5.1 Moxibustion group

**Figure 2:**
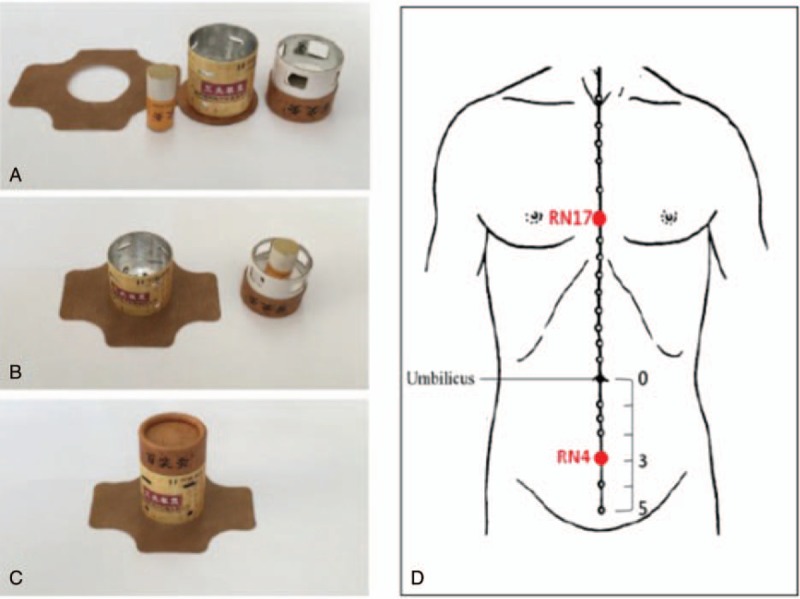
Apparatus-type moxibustion and location of acupoints. (A) From left to right, medical rubberized fabric, moxa cone, moxibustion cylinder, and cap; (B) the moxa cone is fastened onto the cap by the magnet and rubberized fabric is covered onto the moxibustion cylinder; (C) assembled device; (D): location of the RN17 and RN4.

Participants in this group will receive anti-depressant treatment and 14 sessions of moxibustion treatment (over 2 weeks). Apparatus-type moxibustion (Chongqing Happyall Medical Equipment Co., Ltd., specification, BX-A002) will be conducted at two acupoints on the conception vessel meridian, Danzhong (RN17) (located on the anterior midline, on the level of the 4^th^ intercostal space) and Guanyuan (RN 4) (located 3 cm below the center of the umbilicus) for about 20 minutes every day for 2 weeks with the patients lying in supine position. These points were selected according to the clinical experiences of a responsible researcher and literature reviews.^[27,30]^ The moxibustion apparatus composed of the moxibustion cylinder, cap, moxa cone, and medical rubberized fabric. The base of the cylinder has tiny holes that acts as a heat channel. During the operation, an ignited moxa cone will be inserted and fastened onto the cap by a magnet. Then, the cap will be assembled with the cylinder. Finally, the cylinder will be fixed on the acupoint by rubberized fabric. The moxibustion temperature can be controlled by adjusting the air inlet size through horizontal rotation or by lifting the cap up. The apparatus-type moxibustion and location of acupoints are shown in Figure 2.

Moxibustion treatment will be conducted by a licensed Chinese Medicine doctor with more than 10 years of clinical experience to enhance the doctor's adherence to the study protocol and safety in the application of moxibustion.

#### 2.5.2 Wait-list control group

Participants in the wait-list control group will not receive moxibustion treatment during the treatment period. They will maintain their usual anti-depressant treatment.

### 2.6 Follow-up

**Figure 3:**
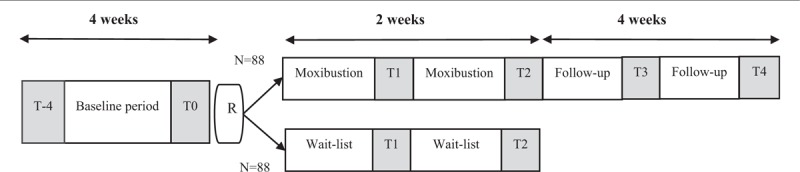
Study timeline. T-4 = study entry, T0 = baseline, T1 = mid-treatment, T2 = post-treatment, T3 and T4 = follow-up assessment, R = randomization.

Only participants in the moxibustion group will be followed-up for 4 weeks (the timeline of this study is shown in Fig. 3).

In this study it is not possible to compare the effects of a moxibustion treatment group and a wait-list control group throughout the whole process of the study, since the deprivation of medicine from patients for a long time would be regarded as unethical and may reduce the compliance of patients as well. Long-term waiting may also lead to drop out. For these reasons, moxibustion treatment will be offered to the wait-list control group, after 2 weeks of waiting, and participants will be evaluated and followed-up exactly in the same manner as the moxibustion group. However, the moxibustion treatment that was offered to the wait-list control group will not be included in the statistical analysis regarding treatment efficacy, and solely the first phase of this study will serve to statistically compare the efficacy of the moxibustion group to wait-list control group.

### 2.7 Outcome measures

At the study entry, baseline data questionnaires including Demographic characteristic, Traditional Chinese Medicine (TCM) pattern identification, etc, will be carefully recorded. All outcome measures and questionnaires will be assessed by assessors and data collectors blinded to the group allocations.

The schedule of enrollment, intervention, and assessments are shown in Table 1.

**Table 1:**
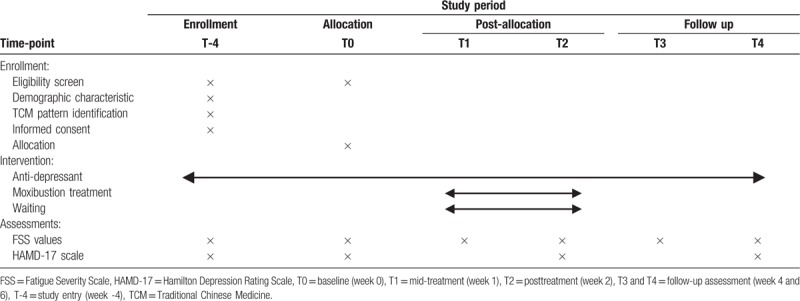
Schedule of enrollment, intervention, and assessments.

#### 2.7.1 Primary outcome measure

The severity of fatigue will be measured by the Fatigue Severity Scale (FSS), which is the most widely used unidimensional fatigue measure that was initially validated in patient groups with multiple sclerosis and systematic lupus erythematosus.^[39]^ Moreover, it also is the most widely used fatigue measure in MDD patients and its psychometric properties have been confirmed in MDD patients.^[40]^ The FSS is a self-reported nine-item questionnaire, that assess fatigue severity and functionality during the past week. Each item is rated on a 7-point Likert scale according to their level of agreement with the given item from 1 (strongly disagree) to 7 (strongly agree). The overall score is the mean of all items’ scores. This FSS will be implemented at baseline, in the 1^st^ and 2^nd^ week after starting treatment, as well as in the moxibustion group's 2^nd^ and 4^th^ week after the end of treatment (week 4 and 6).

#### 2.7.2 Secondary outcome measure

##### 2.7.2.1 Hamilton Depression Rating Scale-17

The assessment of depressive status will be executed with the HAMD-17.^[41]^ The HAMD-17 is the most widely used clinician-administered depression assessment scale and is comprised of 17 items with a three- or five-point scale. Scores will be interpreted as follows: 0 to 7, normal or in clinical remission; 8 to 17, mild depression; 18 to 24, moderate depression; and 25 or over, severe depression. Patients will be assessed with this scale at baseline and at the end of treatment (week 2), as well as in the moxibustion group 4 weeks after the end of treatment (week 6).

##### 2.7.2.2 Safety assessment

To evaluate safety, we will assess the occurrence of AEs related to moxibustion treatment during the study period. All patients will be assessed for signs and/or reports of local and systemic AEs in every visit. The severity of each AE will be classified to mild, moderate, or severe according to Spilker AE classification.^[42]^ All AEs that will be reported by patients and researchers will be recorded in the case report form (CRF) and will be monitored until resolution.

##### 2.7.2.3 Feasibility assessment

The moxibustion treatment will be considered a feasible intervention for patients with depression-related fatigue if at least half of the patients who will be referred to the research center and who have met the eligibility criteria would agree to participate in the study and if the drop-out rate is less than one third.

### 2.8 Data management and quality control

The researchers will be required to follow the requirements of approval protocol and fill all relevant information of the CRF in a timely and accurate manner. The responsible researcher will monitor the clinical trial and quality of the study. The recruitment status, the written informed consent documents, intervention procedures, record of the CRF, timelines of data collection and overall trial progress will be checked by regular monitoring.

### 2.9 Statistical analysis

Statistical analysis will be performed with the use of SPSS 20.0 software (IBM, Armonk, NY) by the statistician, who is independent from the research team and blinded from the groups allocation. The clinical effects of moxibustion on depression related fatigue will be analyzed on an intent-to-treat (ITT) approach. Sensitivity analysis will be conducted to compare the results of the ITT analysis with those of per-protocol (PP) analysis. The PP analysis will include participants who complete at least 80% of the intervention and are assessed for all outcome measures.

Demographic characteristics of participants and baseline measurements of variables will be presented. Continuous data will be reported as mean (standard deviation), while categorical data will be reported as frequencies (percentages). Median and 95% confidence intervals will also be provided if necessary. Repeated measures analysis of variance (ANOVA) will be used for the analysis of variables, which will be measured at several different times. Other variables will be assessed by using one-way analysis of variance (ANOVA) or non-parametric Mann–Whitney *U* test, as appropriate. Paired samples or non-parametric Wilcoxon signed rank tests will be used for the assessment of data in each group. Subgroup analyses will be conducted to determine whether there are significant differences in the responses to moxibustion treatment base on the pre-treatment TCM patterns, or severity of fatigue. *P*-values lower than .05 will be considered statistically significant in this study.

## 3 Discussion

Fatigue is one of the most prevalent and debilitating symptoms of MDD^[3–5]^ and impairs patients’ functioning, abilities and QOL,^[4,9,10]^ and causes a considerable financial burden for society.^[13]^ Therefore, this study is designed to assess the efficacy, safety and feasibility of moxibustion treatment for patients with depression-related fatigue, to develop a comprehensive treatment plan.

### 3.1 Specific fatigue quantifiable scale is necessary for efficacy measures

Fatigue is a complex, multidimensional and subjective experience. Although it is often associated with depression, it is unknown whether it is a distinct entity from depression or whether it can be separately evaluated,^[43]^ which makes a number of assessment challenges.

The complementary use of a fatigue-specific assessment and depression rating scale will provide more advantageous information in the assessment of depression-related fatigue. Therefore, in this study, the FSS will be used as the primary outcome measure to comprehensively assess the symptom of fatigue and the depression status of patients will also be assessed by changes in the HAMD-17 score, which is the study's secondary outcome measure. Studies of fatigue measurement scales have shown that the FSS is the most commonly used fatigue specific questionnaire.^[44]^ Moreover, it is the most widely used fatigue measure in MDD patients and its psychometric properties have been confirmed useful in the evaluation of MDD patients as well.^[40]^ To ensure the stability of data acquisition, the same psychiatrist will assess patients at each point in time.

### 3.2 The influence of Traditional Chinese Medicine patterns on the therapeutic efficacy of moxibustion

The origin of moxibustion can be traced back to the clan commune period of the primitive society in China, with gradual replenishment and development in each generation, moxibustion is now widely applied in treating a great range of diseases.^[45]^ According to TCM, moxibustion is a better option for patients who are categorized into a cold or deficiency pattern.^[26]^ In this study, participants will also be categorized according to their signs and symptoms into patterns such as cold or hot and as deficiency or excess. We will conduct a sub group analysis based on the identified pattern to determine how pre-treatment patterns influence the therapeutic response of moxibustion. The results of the study will help to explore the clinical value of TCM theory concepts and identify patients who are suited for moxibustion therapy.

### 3.3 Advantages

To the best of our knowledge, this trial will be the first study protocol designed to assess the anti-fatigue effect of moxibustion in patients with depression. Given its potential effect, simplicity of application and low cost, moxibustion may be a valuable adjuvant treatment option for many people with depression-related fatigue. Moreover, this method may be especially appealing to patients who do not like the needle penetration sensation associated with acupuncture. The strengths in methodology, including rigorous randomization, and assessors, data collectors and the statistician-blinded, will guarantee the quality of this study. We hope that our data provides a baseline data in this aspect.

### 3.4 Limitations

The main limitation of this study is a lack of objective indicators for assessing the level of fatigue. Especially because fatigue itself is a subjective feeling of the patient, and there is currently no valid and reliable method to objectively evaluate it. Secondly, the lack of a long-term follow-up period in our study makes it impossible to assess the long-term effects of moxibustion on depression-related fatigue. Thirdly, it is not possible to compare the effects of moxibustion on the wait-list control group throughout the whole process of the study, since depriving the patients of medicine for a long-time would be regarded as unethical and would reduce the compliance of patients as well. Finally, although the purpose of this trial is to compare the overall effect of moxibustion to a wait-list control group for depression-related fatigue, a lack of a sham moxibustion group as control may reduce the power of our study to verify the efficacy of moxibustion to a certain degree.

Despite these limitations, the results of this study may provide evidence for the efficacy of moxibustion as an adjunct to antidepressants for depression-related fatigue, and promote a more widespread foundation for the selection of moxibustion in the clinical setting as well as for future research in moxibustion therapy.

## Acknowledgments

The authors would like to thank all of the collaborators and participants of the study.

## Author contributions

**Conceptualization:** Somayeh Iravani, Liwei Cai, Ke Xu, Baixiao Zhao

**Investigation:** Liwei Cai, Shuzhe Zhou, Chuan Shi, Yibin Ma, Ke Xu

**Methodology:** Somayeh Iravani, Liwei Cai, Lue Ha, Shuzhe Zhou, Qin Yao

**Project administration:** Ke Xu, Baixiao Zhao

**Supervision:** Baixiao Zhao

**Writing – original draft:** Somayeh Iravani, Liwei Cai

**Writing – review & editing:** Somayeh Iravani, Liwei Cai, Lue Ha, Shuzhe Zhou, Chuan Shi, Yibin Ma, Qin Yao, Ke Xu, Baixiao Zhao.
